# Leisure Activities and Their Relationship With MRI Measures of Brain Structure, Functional Connectivity, and Cognition in the UK Biobank Cohort

**DOI:** 10.3389/fnagi.2021.734866

**Published:** 2021-11-16

**Authors:** Melis Anatürk, Sana Suri, Stephen M. Smith, Klaus P. Ebmeier, Claire E. Sexton

**Affiliations:** ^1^Centre for Medical Image Computing, Department of Computer Science, University College London, London, United Kingdom; ^2^Department of Psychiatry, Warneford Hospital, University of Oxford, Oxford, United Kingdom; ^3^Nuffield Department of Clinical Neurosciences, Wellcome Centre for Integrative Neuroimaging, Oxford Centre for Functional MRI of the Brain, John Radcliffe Hospital, University of Oxford, Oxford, United Kingdom

**Keywords:** leisure activities, brain, MRI, aging, cognition, UK Biobank

## Abstract

**Introduction:** This study aimed to evaluate whether engagement in leisure activities is linked to measures of brain structure, functional connectivity, and cognition in early old age.

**Methods:** We examined data collected from 7,152 participants of the United Kingdom Biobank (UK Biobank) study. Weekly participation in six leisure activities was assessed twice and a cognitive battery and 3T MRI brain scan were administered at the second visit. Based on responses collected at two time points, individuals were split into one of four trajectory groups: (1) stable low engagement, (2) stable weekly engagement, (3) low to weekly engagement, and (4) weekly to low engagement.

**Results:** Consistent weekly attendance at a sports club or gym was associated with connectivity of the sensorimotor functional network with the lateral visual (β = 0.12, 95%CI = [0.07, 0.18], FDR *q* = 2.48 × 10^–3^) and cerebellar (β = 0.12, 95%CI = [0.07, 0.18], FDR *q* = 1.23 × 10^–4^) networks. Visiting friends and family across the two timepoints was also associated with larger volumes of the occipital lobe (β = 0.15, 95%CI = [0.08, 0.21], FDR *q* = 0.03). Additionally, stable and weekly computer use was associated with global cognition (β = 0.62, 95%CI = [0.35, 0.89], FDR *q* = 1.16 × 10^–4^). No other associations were significant (FDR *q* > 0.05).

**Discussion:** This study demonstrates that not all leisure activities contribute to cognitive health equally, nor is there one unifying neural signature across diverse leisure activities.

## Introduction

By 2050, the total number of older adults (i.e. individuals ≥ 60 years old) worldwide is expected to reach 2.1 billion (Prince et al., 2015). While improvements in life expectancy is a significant achievement of the 21st century, population aging represents a major societal challenge (World Health Organization, 2015). This is because older adults are often at a greater risk of developing certain health conditions than their younger counterparts. For instance, an individual aged 90 or older has 25 times the risk of developing dementia compared to an individual in their late 60s (Yip et al., 2006; Brayne, 2007). Whole population rates of dementia, such as Alzheimer’s disease, are projected to triple by 2050 as a consequence of population aging (Patterson, 2018). Consequently, there has been growing scientific interest in identifying modifiable factors that may reduce the risk of developing dementia and contribute to “better” brain health in late life.

Leisure activities (e.g., visiting friends and family, reading, and going to the cinema or museum) represent one set of modifiable factors that potentially support healthy cognitive aging, by promoting brain plasticity (Stern, 2012). For example, higher activity participation has been systematically linked to better cognitive performance, higher regional and global gray matter (GM) volume, fewer volumetric measures of WM lesions and less decline in the quality of white matter (WM) tracts (Fratiglioni et al., 2004; Wang et al., 2012; Sexton et al., 2013; Erickson et al., 2014; Yates et al., 2016; Anatürk et al., 2018; Chan et al., 2018; Evans et al., 2018; Matyas et al., 2019; Wassenaar et al., 2019). Variability in functional connectivity measures also appears to be partly accounted for by activity engagement, particularly for physically demanding activities (Stillman et al., 2019). However, many of these studies have employed composite measures of leisure activities, providing limited insights into the *specific* activities that need to be targeted to promote brain health in older individuals. Targeting non-optimal activities may in part explain the limited efficacy of current randomized-controlled trials (RCTs) on cognitive and neural outcomes (Mortimer et al., 2012; Stephen et al., 2019).

A small number of epidemiological studies have begun to shift away from the composite approach when examining the link between leisure activities and the aging brain, finding that not all activities equally contribute to the risk of cognitive impairment (Krell-Roesch et al., 2017, 2019; Fancourt et al., 2018). For example, Fancourt et al. (2018) examined data collected from 3,911 participants enrolled in the English Longitudinal Study of Aging and found that adults who visited museums, art galleries and exhibitions on a regular basis, had a lower incidence of dementia over the course of 10 years. The association between cultural activities and dementia incidence appeared to be robust, as it remained significant after adjusting for how frequently these individuals were involved in community-based activities (e.g., social clubs, volunteering, sports clubs), alongside sociodemographic (i.e., age, sex, marital status, education, employment status, wealth, and previous occupational classification), and health-related co-variates (i.e., eyesight, depression, hearing, and existing cardiovascular health conditions). Importantly, a study that aims to evaluate whether different activities relate to markers of brain health requires a comparably larger number of univariate tests, relative to when a single composite measure of leisure activity is of interest. A range of key factors also need to be appropriately adjusted for when investigating the association between leisure activities and the aging brain, due to the bias that these confounding variables can otherwise introduce in the results and conclusions of a study, including inflated effect sizes or even spurious findings. For example, education and socio-economic status (SES) are determinants of how often a person engages in cultural activities such as visiting a museum, art gallery and exhibition (Grisolía et al., 2010; Mak et al., 2020). As low levels of education and SES are linked to an increased risk of dementia (Cadar et al., 2018; Livingston et al., 2020), demonstrating an independent relationship between cultural activities and the dementia risk strengthens the argument that these activities potentially contribute to maintaining brain health in older ages. The results of such correlational studies can then help guide randomized controlled trials (RCTs) to focus their intervention programs on a specific subset of activities, to determine whether these reported associations translate to direct effects on the aging brain. With a sample that currently exceeds several thousand well-characterized individuals, the United Kingdom Biobank (UK Biobank) study offers the statistical power to employ a more fine-grained approach to investigate the relationship between activities and markers of brain health, after adjusting for a wide range of confounders. This cohort study also offers measures of activity levels at more than one timepoint, allowing us to capture how longitudinal patterns of activities relate to metrics of brain health.

The aim of this study was to investigate whether individual leisure activities relate to MRI measures of GM volume, WM microstructure, WM lesions, resting-state functional connectivity, and cognitive function in late life. We examined six leisure activities that were available in the UK Biobank: going to a pub or social club, undertaking a religious activity, attending adult education classes, going to a sports club or gym, visiting friends and family and leisure-time computer use. Based on their self-reported activity levels at two timepoints, individuals were divided into one of four groups: (1) stable weekly participation, (2) stable low participation, (3) low to weekly participation, and (4) weekly to low participation. We predicted that weekly activity participation (both stable and increased participation over time) would associate with higher global cognitive function (Brown et al., 2012; Mitchell et al., 2012; Wang et al., 2012; Yates et al., 2016; Evans et al., 2018). We further expected that consistently high or increased participation in each leisure activity would correlate with greater structural integrity, including greater regional GM and higher WM integrity (i.e., higher fractional anisotropy (FA), lower mean diffusivity (MD; Anatürk et al., 2018). Given the limited evidence investigating activity-specific effects on resting-state functional connectivity, no predictions were made regarding this modality, so any results would be exploratory.

## Materials and Methods

### Sample Characteristics

Data was provided by participants enrolled in the UK Biobank study, a large-scale prospective cohort study. These individuals were asked to complete a range of assessments including detailed lifestyle questionnaires, cognitive tests, physical measures (e.g., blood pressure and mobility tests), provide biological samples (e.g., blood, urine, and saliva) and also provide permission to access their National Health Services (NHS) health records. Since 2014, a sub-sample of the original 500,000 participants have been invited back to undergo a single session of MRI scanning of the brain, body and heart, with the goal of reaching 100,000 scanned individuals by 2022. Their recruitment continues, with regular data releases made available to researchers (Miller et al., 2016). At the time of paper preparation, imaging data from a total 15,000 participants had been released (January 2019).

In our analyses, we examine data collected from two study phases: at recruitment (2006–2010) and MRI assessment (2014+). As demonstrated in Supplementary Figure 1, leisure activity measures were taken from both timepoints, while MRI and cognitive data was taken at the second timepoint (mean years between timepoints = 8 years, range = 4–11 years). The UK Biobank study received ethical approval from the NHS National Research Ethics Service (Ref 11/NW/0382) and all enrolled participants gave their informed and written consent.

### Inclusion and Exclusion Criteria

The sample consisted of individuals without a diagnosis of stroke or dementia for the study duration, who had completed an MRI assessment and provided complete data on leisure activities and sociodemographic, health, cognitive and lifestyle variables (for flowchart, see Supplementary Figure 2).

### Activities

Activity levels were assessed through items displayed on a touch screen tablet. From a list of activities, participants were required to highlight those that they participated in on at least a weekly basis. Accordingly, we coded response option for each activity as (1) weekly or (2) less than weekly participation. A total of six activities were examined, which included going to a pub or social club; undertaking a religious activity; attending adult education classes; going to a sports club or gym; visiting friends and family and leisure-time computer use. Note that visiting friends and family and computer use were measured with different response options (i.e., frequency and hours) and these items were binarized into weekly/less than weekly engagement to harmonize the scales with the remaining activity measures. Further information on these items is provided in the Supplementary Methods section “Activity Measures*.”* Individuals were then split into one of four groups: (1) weekly stable engagement, (2) low stable engagement, (3) low to weekly engagement, and (4) weekly to low engagement.

### Cognitive Function

A 15-min study-specific battery of cognitive assessments was administered to participants via a touch screen tablet (Cornelis et al., 2019). The cognitive measures examined are described in Table 1, with a detailed description of each measure in the Supplementary Material. A measure of global cognitive function was computed by summing across all cognitive measures. Prior to this step, all continuous cognitive scores were standardized, with the outcomes of the alphanumeric and numeric trail making tasks and Pairs Matching test reverse scored to allow higher scores to reflect “better” performance.

**TABLE 1:** Neuropsychological tests of the UK Biobank battery examined in the present study.

Cognitive test	Outcome
Fluid intelligence	Total number of questions answered correctly (maximum score: 13)
Numeric/Alphanumeric Trail making	Time (in seconds) taken to complete the trail
Digit span	Maximum number of digits recalled (maximum score: 12)
Pairs matching	Number of incorrect matches made
Prospective memory	Whether or not participant responded correctly at first attempt
Symbol digit matching	Number of correct symbol-digit matches
Simple reaction time	Mean response time (in seconds) across the 4 trials containing matching pairs

### Demographic and Health-Related Variables

Baseline measures of age, sex, education, occupation, frequency of alcohol intake, sleep duration, body mass index (BMI), Mean arterial pressure (MAP), and social isolation (total number of individuals in household) were collected. ICD-10 diagnoses of depressive or anxiety disorders developed over the study duration was also examined. Additional information about these variables can be found in the Supplementary Material.

### MRI Data Acquisition and Pre-processing

Participants were scanned using identical protocols with Siemens Skyra 3T (software VB13) and a Siemens 32-channel head coil at one of two study sites (i.e., Stockport or Newcastle).

T_1_-weighted images, diffusion-weighted images, T2 FLAIR images and resting-state functional images were assessed. Summary measures of brain structure and functional connectivity, or Image Derived Phenotypes (IDPs), have been generated on behalf of UK Biobank (Alfaro-Almagro et al., 2018) and are available from UK Biobank upon data access application. For a detailed description of the imaging protocol and pre-processing steps, please see the Supplementary Material. The IDPs generated from this pipeline consisted of total and regional GM volume (142 IDPs), total WM volume and lesions within WM (2 IDPs), WM microstructure (FA and MD in 27 pre-defined tracts, 54 IDPs), and partial correlation functional connectivity between large-scale resting-state networks (210 IDPs). GM and WM IDPs were averaged across left and right hemispheres, resulting in a total of 76 GM outcomes and 63 WM outcomes. For a comprehensive list of all IDPs examined in our study, please see Supplementary File: List of MRI Outcomes.

### Statistical Analysis

All analyses were performed in R (version 3.5.2). The lm function in R was used to fit a series of linear models to evaluate the relationship between each type of activity (e.g., going to a sports club or gym; visiting friends and family) and each of the neuroimaging outcomes and global cognition, after adjusting for a range of co-variates. The co-variates included age, sex, education, occupational status, assessment center, BMI, Mean Arterial Pressure (MAP), frequency of alcohol intake, sleep duration, the presence of depressive or anxiety disorders and the number of individuals living in a household. Mean head motion and head size were also included as co-variates in the analysis of neuroimaging metrics. Six linear models (one for each type of activity) were run across the entire sample for every activity-outcome combination, with 139 MRI outcomes (i.e., 76 GM and 63 WM measures) and 1 cognitive outcome examined. This resulted in a total of 840 linear regressions that were conducted, with an example of the general formulae used for these models provided below:

y = β0 + β1 weekly stable activity engagement + β2 low to weekly activity engagement + β3 weekly to low activity engagement + β4 age + β5 sex + β6 education + β7 occupation + β8 assessment center + β9 body mass index + β10 mean arterial pressure + β11 alcohol intake + β 12 sleep duration + β13 depressive/anxiety disorder + β14 number in household + β15 mean head motion (MRI outcomes only) + β16 head size (MRI outcomes only) + ε.

Prior to the analysis, the distributions of all dependent variables were screened, with the distributions of total WM lesions log-transformed due to non-normality. All continuous variables were also standardized before the analysis, while categorical variables were dummy coded. A total of three dummy variables were used to reflect different engagement patterns for a given activity: (1) weekly stable activity engagement (=1, all other groups = 0), (2) low to weekly activity engagement (=1, all other responses = 0), and (3) weekly to low activity engagement (=1, all other responses = 0). Based on prior evidence indicating that low levels of activity are linked to poorer brain health and cognitive outcomes (Brown et al., 2012; Mitchell et al., 2012; Wang et al., 2012; Yates et al., 2016; Anatürk et al., 2018), individuals in the “low stable” group were chosen as our reference category. Comparisons of age, sex and education and ICD-10 diagnosis of anxiety/depression between each group for a given activity can be found in Supplementary File: Group Comparisons.

Due to the number of univariate tests conducted, FDR-corrections were applied. To facilitate these corrections, the p.adjust function in R was applied with a two-tailed FDR *q* value < 0.05 considered significant (Cox et al., 2019). We report standardized beta coefficients (β), 95% confidence intervals and FDR *q*-values in the main text. The results of all associations examined are also included in the Supplementary File: Results. Excluded and included participants were compared on age, sex, education and ICD-10 diagnosis of anxiety or depression, which are reported in Supplementary Table 1.

## Results

### Participant Demographics

Table 2 provides a detailed description of the sample demographics. In brief, a total of 7,152 participants were included in the analysis of neuroimaging outcomes. Participants were on average 56.39 years old (*SD* = 7.31) at baseline and 63.94 years old (*SD* = 7.32) at follow-up. Females represented 54.5% of the sample (*n* = 3,897). A substantially smaller number of individuals had provided complete data for all cognitive measures at follow-up and were therefore analyzed as a sub-set (*n* = 1,734) of the main sample. The percentage of participants in each activity group (along with the percentage of females) are reported in Table 3. Comparisons between the analytical samples and excluded participants (Supplementary Table 1) suggested that the MRI sample were significantly older, more educated and had a higher occupation status and higher proportion of females than compared to excluded participants. These group differences were also found for sex, education and occupational status in the cognitive sample. No significant group differences were observed in the proportion of individuals with an ICD-10 diagnoses of anxiety/depression in either sample. For group comparisons on these variables between each activity group, please see Supplementary File: Group Comparisons.

**TABLE 2:** Sample characteristics.

		Range
No. of participants	7,152	
Duration between baseline and MRI scan (years)	7.55	4.29–10.85

**Demographics**		

Age at baseline (years)	56.39 ± 7.31	40–70
Age at MRI scan (years)	63.94 ± 7.32	46–80
No. of females (%)	3,897 (54.5)	
Highest educational qualification (%)	O levels/GCSE = 146 (2%) | CSE = 771 (10.8%) | A levels/AS = 398 (5.6%) NVQ/HND/HNC = 2213 (30.9%) | CU = 3624 (50.7%)	
Occupational status n. (%)	0 = 1480 (20.7%) | 1 = 17 (0.2%) | 2 = 29 (0.4%) | 3 = 37 (0.5%) | 4 = 58 (0.8%) | 5 = 154 (2.2%) | 6 = 513 (7.2%) | 7 = 1046 (14.6%) | 8 = 2290 (32%) | 9 = 1528 (21.4%)	

**Activities**		

**No. (%) reporting weekly participation at**	**Baseline**	**Follow-up**

Leisure-time computer use	6,340 (88.7)	6,778 (94.8)
Visiting friends and family	5,750 (80.4)	5,874 (82.1)
Going to the pub or social club	2,540 (35.5)	2,513 (35.1)
Undertaking religious activities	1,723 (24.1)	1,770 (24.8)
Attending educational courses	903 (12.6)	775 (10.8)
Going to a sports club or gym	3,852 (50.1)	3,539 (49.5)

**Health and Lifestyle**		**Range**

BMI (kg/m^2^)	26.4 ± 4.01	16.14–55.07
MAP	99.57 ± 11.84	60–152.83
No. (%) with ICD-10 diagnosis of Depression/Anxiety	51 (0.71)	
Sleep duration (hours/night)	7.21 ± 0.93	2–13
Alcohol (frequency/week)	0 = 300 (4.2%) | 1 = 499 (7%) | 2 = 664 (9.3%) | 3 = 1,837 (25.7%) | 4 = 2,183 (30.5%) | 5 = 1,669 (23.3%)	

**Structural MRI measures**		

Total GM (volume, mm^3^)	613,301.98 ± 54,614.44	443,926–832,927
WM (volume, mm^3^)	547,421.16 ± 61,214.82	362,561–804,641
CSF (volume, mm^3^)	36,479.39 ± 17,032.15	7,613.27–157,075
WM hyperintensities (volume, mm^3^)*	2,752 (4054.5)	30–86,534

**Cognitive function (*n* = 1,734)**		

Fluid intelligence score	7.04 ± 1.93	1–13
Alphanumeric trail making (seconds)*	47.9 (20.08)	21.10–242.10
Numeric trail making (seconds)*	19.9 (6.5)	9.40–112.90
Pairs matching test (total errors)*	6 (4)	0–28
Simple reaction time (seconds)	0.59 ± 0.1	0.37–1.34
No. of correct symbol-digit matches	19.74 ± 5.14	2–36
Backward digit span score	6.91 ± 1.22	2–12
Prospective memory score (No. % correct)	1,510 (87.1%)	

Values are Mean ± Standard deviation and N (%) for categorical variables, unless otherwise stated. *Median (IQR) reported. BMI, body mass index; BP, blood pressure; CSE, certificate of secondary education, CSF, cerebrospinal fluid; CU, college or university degree, GCSE, general certificate of secondary education; GM, gray matter; HNC, higher national certificate; HND, higher national diploma; ICD, international classification of diseases; MAP, mean arterial pressure; MRI, magnetic resonance imaging; OL, O-levels, N, number; NVQ, national vocational qualifications; WM, white matter. Occupational status was coded according to the following scale: 0 = “Unemployed/Retired/Unable to work/Student,” 1 = “Elementary Occupations,” 2 = “Process, plant and machine operatives,” 3 = “Sales and Customer Service Occupations,” 4 = “Personal Service Occupations,” 5 = “Skilled Trades Occupations,” 6 = “Administrative and Secretarial Occupations,” 7 = “Associate Professional and Technical Occupations,” 8 = “Professional Occupations,” and 9 = “Manager and Senior Officials. Alcohol intake was measured on a 5-point scale: 5 = “daily or almost daily,” 4 = “three or four times a week,” 3 = “once or twice a week,” 2 = “one to three times a month,” and 1 = “special occasions only” or 0 = “never.”

**TABLE 3:** Summarizing the number of individuals in each activity group, for the MRI and cognitive samples.

Activity	Stable low engagement	Weekly to low engagement	Low to weekly engagement	Stable weekly engagement
**MRI sample**				
**Computer use** *No. of females (%)*	197 (2.8%); 129 (65.5%)	177 (2.5%); 120 (67.8%)	615 (8.6%); 412 (67%)	6163 (86.2%); 3,236 (52.5%)
**Educational classes** *No. of females (%)*	5804 (81.2%); 2,947 (50.8%)	573 (8%); 387 (67.5%)	445 (6.2%); 312 (70.1%)	330 (4.6%); 251 (76.1%)
**Religious activities** *No. of females (%)*	5,208 (72.8%); 2,659 (51.1%)	174 (2.4%); 106 (60.9%)	221 (3.1%); 152 (68.8%)	1,549 (21.7%); 980 (63.6%)
**Pub/social club** *No. of females (%)*	3974 (55.6%); 2,573 (64.7%)	665 (9.3%); 347 (52.2%)	638 (8.9%); 340 (53.3%)	1875 (26.2%); 637 (34%)
**Sports club/gym** *No. of females (%)*	2707 (37.8%); 1,408 (52%)	906 (12.7%); 529 (58.4%)	875 (12.2%); 525 (60%)	2664 (37.2%); 1,435 (53.9%)
**Visiting friends/family** *No. of females (%)*	685 (9.6%); 314 (45.8%)	593 (8.3%); 277 (46.7%)	717 (10%); 377 (52.6%)	5157 (72.1%); 2,929 (56.8%)

**Cognitive sample**				

**Computer use** *No. of females (%)*	45 (2.6%); 31 (68.9%)	48 (2.8%); 36 (75%)	142 (8.2%); 89 (62.7%)	1,499 (86.4%); 795 (53%)
**Educational classes** *No. of females (%)*	1,385 (79.9%); 704 (50.8%)	161 (9.3%); 107 (66.5%)	110 (6.3%); 83 (75.5%)	78 (4.5%); 57 (73.1%)
**Religious activities** *No. of females (%)*	1,270 (73.2%); 643 (50.6%)	44 (2.5%); 27 (61.4%)	45 (2.6%); 34 (75.6%)	375 (21.6%); 247 (65.9%)
**Pub/social club** *No. of females (%)*	958 (55.2%); 638 (66.6%)	162 (9.3%); 87 (53.7%)	169 (9.7%); 82 (48.5%)	445 (25.7%); 144 (32.4%)
**Sports club/gym** *No. of females (%)*	621 (35.8%); 325 (52.3%)	208 (12%); 119 (57.2%)	241 (13.9%); 137 (56.8%)	664 (38.3%); 370 (55.7%)
**Visiting friends/family** *No. of females (%)*	167 (9.6%); 73 (43.7%)	155 (8.9%); 73 (47.1%)	184 (10.6%); 92 (50%)	1,228 (70.8%); 515 (41.9%)

*Numbers and percentages are reported as well as the percentage of females in each group. For comparisons of age, sex, education and the presence of ICD-10 diagnoses of anxiety/depression, please see Supplementary File: Group Comparisons.*

### Cognitive Function

Stable weekly computer use was linked to higher global cognitive performance, relative to stable low computer use (β = 0.62, 95%CI = [0.35, 0.89], FDR *q* = 1.16 × 10^–4^). No other activities were significantly linked to cognitive performance (FDR *q*’s > 0.05).

### Structural MRI

Stable weekly family/friend visits were associated with higher GM volume in the occipital pole, in comparison to individuals consistently reporting infrequent family and friend visits (β = 0.15, 95%CI = [0.08, 0.21], FDR *q* = 0.03). No significant associations were found for any other activity with GM volume, tract-specific FA or MD, total WM volume or lesions (FDR *q*’s > 0.05).

### Functional MRI

**FIGURE 1:**
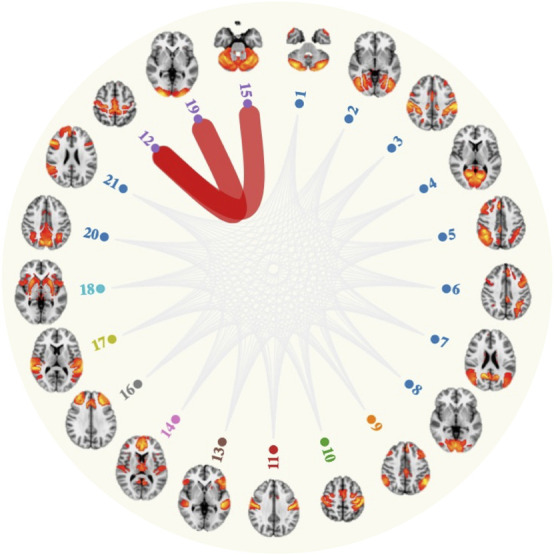
Attending a sports club and gym on a weekly basis associated with stronger connectivity (red lines) between the sensorimotor network (node 12) and lateral visual network (node 19) and cerebellar network (node 15).

Stable weekly engagement at a sports club or gym was associated with stronger absolute connectivity between the sensorimotor network and lateral visual network, relative to stable low engagement (β = 0.12, 95%CI = [0.07, 0.18], FDR *q* = 2.04 × 10^–2^; Figure 1). Similarly, stronger connectivity was also observed between the sensorimotor and cerebellar networks for this activity (β = 0.12, 95%CI = [0.07, 0.18], FDR *q* = 2.04 × 10^–2^; Figure 1). The association for sensorimotor-cerebellar network connectivity (β = 0.12, 95%CI = [0.07, 0.18], FDR *q* = 1.23 × 10^–4^) and sensorimotor-lateral visual network connectivity (β = 0.12, 95%CI = [0.07, 0.18], FDR *q* = 2.48 × 10^–3^) remained significant after adjusting for physical activity levels (i.e., summed weekly Metabolic equivalent of task (MET) minutes, n. with physical activity information = 6,289) and global GM volume. There were no significant associations between any other activity and resting-state functional connectivity strength (FDR *q* > 0.05).

## Discussion

We present the largest multi-modal study to examine whether patterns of engagement in leisure activities associate with markers of brain structure, functional connectivity, and cognition. We found that stable weekly participation in a sports club or gym correlated positively with connectivity of the sensorimotor network. Additionally, weekly visits to or from family and friends was associated with higher volumetric measures of GM in the occipital pole. However, apart from consistent leisure time computer use, no other activity was significantly linked to global cognition. Each of these results are discussed, in turn.

One of our main findings was that consistent, weekly visits to a gym or sports club correlated with greater absolute connectivity of the sensorimotor network and cerebellar network, relative to infrequent attendance over time. Similar activation patterns at rest and during task execution indicate that both the sensorimotor and cerebellar networks play a role in motor tasks (Smith et al., 2009), potentially interacting with one another to support motor behavior. Subdivisions of the cerebellum are typically found within the sensorimotor network (Habas et al., 2009; Dobromyslin et al., 2012), an observation that was also replicated in our study (Supplementary Figure 3). The present findings additionally identified a link between connectivity of the sensorimotor and lateral visual network in individuals reporting consistent gym and sports club attendance across time, suggesting that sustained activity engagement contributes to enhanced visual-motor coupling. Potentially underlying this relationship is the importance of visual information in regulating and updating current behavior during physical exercise (Mallek et al., 2017). Our findings are in line with a previous study reporting strengthened connectivity between regions involved in the sensorimotor network, cerebellar and visual network, following a 13-week cognitive and motor skills training intervention (Demirakca et al., 2016). Task based fMRI has also highlighted a greater BOLD response in regions involved in these networks (Domingos et al., 2021). For example, the supplementary motor areas, precentral gyrus, angular gyrus and supramarginal gyrus were among the areas to demonstrate greater BOLD activation during a famous face recognitions task, in a group of adults (age range = 65 to 85) reporting high physical activity levels (*n* = 34) relative to a low physical activity group (*n* = 34, Smith et al., 2011). However, stronger connectivity *between* resting-state networks in an aging sample is not entirely straightforward to interpret. For instance, older adults often exhibit less segregated sensorimotor networks (i.e., lower within-network connectivity and higher between-network connectivity) when compared to younger adults, with these increases in connectivity linked to poorer task performance (e.g., reaction time, dexterity; Cassady et al., 2019). Additionally, physical activity levels did not account for the associations observed across the sensorimotor-visual-cerebellar networks, suggesting that other factors (e.g., physical fitness or socially interacting with others) may support these associations.

A difficulty in interpreting our findings is that while gym/sports club attendance was linked to greater connectivity between the visual and sensorimotor networks, no parallel associations were found between this activity and cognition, despite an established link between physical activity participation and cognitive function (Kelly et al., 2014; Erickson et al., 2019). The absence of a relationship with global cognition may be partly explained by the lower statistical power present in the analyses of cognitive function relative to the MRI analyses, due to the smaller sample size (1,734 individuals versus 7,152 individuals in the MRI sample). As we focused our analyses on global cognition, our study may have also failed to detect specific cognitive domains that are sensitive to gym/sports club participation (e.g., executive function (Erickson et al., 2019). Future studies examining cognitive subdomains could therefore provide novel insights into the cognitive correlates of these types of activities. Additionally, we were only able to generate a crude measure of activity participation based on the measures available in the UK Biobank study, which potentially hampered our ability to detect a link between gym/sports club attendance and cognitive function. Specifically, the granularity of our activity measure may have prevented us from capturing sufficient inter-individual variation in activity levels, such as between individuals who attended a gym/sports club once a week to those who attend it on a more regular basis (e.g., five times a week). Further work would therefore benefit from using more fine-grained assessments of activities (e.g., duration and frequency), as this approach could offer enhanced sensitivity in detecting associations between gym/sport club attendance and cognitive function. A final and more general consideration is that attending the gym or sports clubs is unlikely to serve as an accurate proxy of overall physical activity levels, as many activities can be performed outside of these settings (e.g., running and cycling). This could explain why our study failed to replicate well-established links between physical activity, GM and WM outcomes (Cheng, 2016; Sexton et al., 2016; Wassenaar et al., 2019).

Another of our key findings was that consistently visiting friends and family on a weekly basis associated with higher GM volume in the occipital pole, relative to low and stable patterns. This finding offers partial support for our second hypothesis. Two previous studies have also reported a relationship between higher social activity levels and higher GM volume within this region (James et al., 2012; Arenaza-Urquijo et al., 2016), consistent with the “brain maintenance” (Nyberg et al., 2012) or “brain reserve” (Stern, 2012) hypotheses. However, other studies have not replicated this association (Foubert-Samier et al., 2012; Seider et al., 2016; Anatürk et al., 2020), or otherwise report associations across frontal, parietal and temporal regions (Valenzuela et al., 2008; Arenaza-Urquijo et al., 2015; Seider et al., 2016). The discrepant findings may be due to the use of a cross-sectional assessment of activities in most of these studies or alternatively a lack of assessment into activity-specific relationships. While the frontal and temporal regions are considered to be the most age-sensitive, GM in the occipital cortex also demonstrates a negative relationship with age (e.g., Allen et al., 2005; Fjell and Walhovd, 2010; Lemaitre et al., 2012; Raz et al., 2005). Sustained interpersonal interactions could potentially prevent age-related neuronal or synaptic loss or otherwise promote synaptic or dendritic plasticity (Anatürk et al., 2018). Animal studies offer support for this interpretation (e.g., Briones et al., 2004; Okuda et al., 2009; Zhao et al., 2011; Jung and Herms, 2014), with one study reporting an increase in the synaptic density of the rat visual cortex after housing in an enriched environment for a month (Briones et al., 2004). At the same time, the selectivity of the occipital cortex needs to be interpreted with caution, given the “blunt” nature of our activity measure. Overall, our results suggest that encouraging middle-aged and older adults to stay engaged with their existing social network could potentially be an important avenue for supporting individuals through their later years of life.

Individuals who consistently reported weekly computer use had higher global cognitive performance, relative to those who consistently reported less frequent participation over time. Despite the small number of individuals in the low and stable group (i.e., reference group; *n* = 197) this association was one of the few to survive FDR corrections. These results are in line with prior findings indicating a link between frequent computer (Tun and Lachman, 2010) and internet use (Berner et al., 2019) and better cognitive function. Our findings further complement the observation that computerized cognitive training programs lead to improvements in trained cognitive domains, with some findings suggesting transfer to untrained domains (e.g., Kueider et al., 2012; Nguyen et al., 2019). While speculative, the pathway linking computer use to cognition could be through an increased exposure to novelty (e.g., reading articles online), a greater demand on psychomotor skill (through mouse use and typing) and/or the opportunity to engage several domains of cognition at once (e.g., attention and memory when playing computer games; Tun and Lachman, 2010). Nevertheless, we cannot rule out the explanation that individuals who frequently use the computer are more accustomed to using technology and may have had an advantage to non-users on the computerized cognitive assessments administered in the UK Biobank. Reverse causation could alternatively explain our results, such that individuals with higher levels of cognitive function are inclined to spend more of their time using computers relative to individuals with lower levels of global cognition. While no structural or functional correlates were found for this activity, a link with GM volume in the putamen was detected at trend-level (β = 0.22, 95%CI = [0.12, 0.33], FDR *q* = 0.07), Supplementary File: Results). The putamen is implicated in both movement preparation and execution and non-motor functions, (e.g., executive control, working and episodic memory and category fluency, Ell et al., 2011) and although the trend reported here needs to be independently validated by future studies, it could represent a potential region of interest for studies investigating potential mediators of the computer-cognition association. Taken together, our results suggest that improving computer use level among middle-aged adults represents an important aim of future RCTs, which will also concurrently establish the directionality of the associations reported here.

Otherwise, contrary to our hypotheses, none of the other activities examined (i.e., going to the pub or social clubs, attending an adult educational class, undertaking religious activities) were associated with markers of GM, WM microstructure, functional connectivity or cognitive function, after FDR corrections. While prior meta-analytic investigations have identified associations with brain structure (Anatürk et al., 2018) and cognition (Kuiper et al., 2016) when composite measures of activities were used, our results suggest that comparatively speaking, they do not uniquely contribute to brain health in early late life. The alternative explanation is that the crude measure of activity levels used in our study potentially attenuated associations between these activities and neural/cognitive outcomes. Therefore, an important next step for this line of work is to examine whether our results are replicated when assessing other dimensions of engagement (i.e., duration and frequency). Considering that the present findings imply dissociable effects between activities in brain-cognition associations, our results are in favor of an approach sensitive to these inter-activity differences when the statical power of a study allows for it. However, it also needs to be considered that cumulative engagement over separate activities is likely necessary for sustained improvement in brain function, especially as only weak associations were found for the individual activities investigated in our study. Accordingly, we repeated our results with the inclusion of cumulative leisure activities (i.e., number of activities engaged in on a weekly basis across the two timepoints) and found that although cumulative activities were not significantly linked to any MRI or cognitive outcomes, a trend (i.e., β = 0.03, 95%CI = [0.02, 0.05], FDR *q* = 0.066, Supplementary File: Results [Posthoc MRI results]) was detected for higher cumulative activity and stronger connectivity between the medial visual and posterior default mode network, which was not observed for any specific activity. Hence, activity-specific associations could serve as complementary to that of composite leisure activity scores. Overall, our results are informative to clinicians and researchers planning intervention studies as we highlight several activities that may play a role in maintaining brain health in older adult populations.

### Strengths and Limitations

The core strength of this study is the use of longitudinal leisure activity data provided by a large cohort of middle-aged and older adults. This design enabled novel insights into the neural and cognitive correlates of life activities, while minimizing the risk of recall errors inherent in retrospective assessments of baseline activity levels (Gow et al., 2017). Furthermore, corrections for multiple comparisons were applied to minimize the risk of type I errors, due to the large number of associations examined. However, we were only able to examine six activities due to limited coverage in the Biobank study. Other common activities, such as reading (Paillard-Borg et al., 2009) were not investigated. There was also a lack of specificity in some of the activity items, for example, no information was collected on the type of social or sports clubs that respondents attended. These highlighted weaknesses in our activity measure reflect a more general limitation in the field, where activity questionnaires are often brief or otherwise include non-specific items on club participation (e.g., Cognitive Activities Scale (Wilson et al., 1999, 2003, 2005); Cognitive Reserve Index Questionnaire (Nucci et al., 2012); Cognitive & Leisure Activity Scale (Galvin et al., 2021). We therefore recommend that the activity measures employed by future studies include a comprehensive list of activities and integrate an open-ended question that allows respondents to clarify the type of sports/social clubs that they participate in during their leisure time.

The observational design is also a major weakness of this study. Due to the opportunistic self-selected nature of the sample, we are unable to rule out reverse causation or residual confounding by a third unaccounted for variable. Additionally, at the time of manuscript preparation only a single timepoint of MRI data was available, meaning that the associations reported between gym/sports club attendance and connectivity of the sensorimotor and lateral network could reflect *pre-existing* differences in visual-motor coupling between individuals who engage frequently in this activity compared to those who do so infrequently. Future releases of longitudinal MRI data from the UK Biobank study will help to delineate the directionality of these associations and RCTs will serve to establish whether the reported associations translate to direct effects. Cohort effects serve as another alternative explanation of our results. For example, while older individuals who are now fully engaged with technology may gain cognitive benefits, the same effects might not be observed in 20 years’ time as it becomes more common for individuals to become computer literate from a young age. We also note significant differences between individuals included in the sample to those excluded, with those included generally being older, more educated and from a higher occupational grade and more likely to be female. These comparisons complement the observation that the larger Biobank cohort is not entirely representative of the British general population (Fry et al., 2017). This would suggest that our results are most applicable to those who share similar characteristics to our sample and may not equally generalize to all middle- and older-aged adults.

A final limitation of our study is that while we compared each trajectory group against the “low and stable” group for each activity, we did not compare these trajectory groups against each other, which may have revealed further insights into how differences in activity trajectories relate to brain structure, functional connectivity and cognition. This study therefore represents an initial step toward better characterizing activity-specific associations with the brain but is by no means exhaustive. Further work is required to parse out the specific set of activities that have greater implications for brain aging, generating evidence that may help improve current RCTs designs and retirement programs to ensure that the most promising activities are targeted.

## Conclusion

We found that sustained sports club/gym attendance was linked to greater absolute connectivity of the sensorimotor connectivity although no parallel associations with cognition were found. Conversely, consistent family and friend visits over time were associated with higher volumetric measures of GM in the occipital cortex. Only weekly leisure time computer use over time was linked higher levels of global cognition. Overall, this study demonstrates selective associations between different leisure activities, highlighting several that may be relevant for RCTs aiming to promoting cognitive health in late life.

## Data Availability Statement

The data analyzed in this study is subject to the following licenses/restrictions: Data access to the UK Biobank will need to requested through a standard data access procedure. Requests to access these datasets should be directed to http://www.ukbiobank.ac.uk/register-apply.

## Ethics Statement

The studies involving human participants were reviewed and approved by NHS National Research Ethics Service (Ref 11/NW/0382). The patients/participants provided their written informed consent to participate in this study.

## Author Contributions

SMS provided the overall scientific strategy for UK Biobank brain imaging. MA planned and conducted the analyses and prepared the manuscript, including all tables and the figures. SS, SMS, CES, and KPE provided feedback and comments on all versions of the manuscript. All authors contributed to the article and approved the submitted version.

## Conflict of Interest

This research was conducted using the UK Biobank Resource under the approved application of 45301. MA was supported by the Clarendon Trust DPhil Fellowship and HDH Wills 1965 Charitable Trust (1117747). SS reports funding from the Academy of Medical Sciences/the Wellcome Trust/the Government Department of Business, Energy and Industrial Strategy/the British Heart Foundation/Diabetes UK Springboard Award (SBF006\1078). SMS receives support from the Wellcome Trust (098369/Z/12/Z, 203139/Z/16/Z). KPE reports support from the UK Medical Research Council (G1001354, MR/K013351/), the HDH Wills 1965 Charitable Trust (1117747), Alzheimer’s Research UK (PPG2012A-5), and the European Commission (Horizon 2020 grant “Lifebrain,” 732592). SS and CES were supported by the NIHR Oxford Biomedical Research Center located at the Oxford University Hospitals NHS Trust and the University of Oxford, the NIHR Oxford Health BRC. The Wellcome Center for Integrative Neuroimaging was supported by core funding from the Wellcome Trust (203139/Z/16/Z). CES is now a full-time employee of the Alzheimer’s Association.

## Publisher’s Note

All claims expressed in this article are solely those of the authors and do not necessarily represent those of their affiliated organizations, or those of the publisher, the editors and the reviewers. Any product that may be evaluated in this article, or claim that may be made by its manufacturer, is not guaranteed or endorsed by the publisher.

## References

[B1] Alfaro-Almagro F. Jenkinson M. Bangerter N. K. Andersson J. L. R. Griffanti L. Douaud G. (2018). Image processing and quality control for the first 10,000 brain imaging datasets from UK Biobank. *Neuroimage* 166 400–424. 10.1016/J.NEUROIMAGE.2017.10.034 29079522 PMC5770339

[B2] Allen J. S. Bruss J. Brown C. K. Damasio H. (2005). Normal neuroanatomical variation due to age: the major lobes and a parcellation of the temporal region. *Neurobiol. Aging* 26 1245–1260. 10.1016/j.neurobiolaging.2005.05.023 16046030

[B3] Anatürk M. Demnitz N. Ebmeier K. P. Sexton C. E. (2018). A systematic review and meta-analysis of structural magnetic resonance imaging studies investigating cognitive and social activity levels in older adults. *Neurosci. Biobehav. Rev.* 93 71–84. 10.1016/J.NEUBIOREV.2018.06.012 29940239 PMC6562200

[B4] Anatürk M. Suri S. Zsoldos E. Filippini N. Mahmood A. Singh-Manoux A. (2020). Associations between longitudinal trajectories of cognitive and social activities and brain health in old age. *JAMA Netw. Open* 3:e2013793. 10.1001/jamanetworkopen.2020.13793 32816032 PMC7441365

[B5] Arenaza-Urquijo E. M. de Flores R. Gonneaud J. Wirth M. Ourry V. Callewaert W. (2016). Distinct effects of late adulthood cognitive and physical activities on gray matter volume. *Brain Imag. Behav.* 11 346–356. 10.1007/s11682-016-9617-3 27757821

[B6] Arenaza-Urquijo E. M. Wirth M. Chételat G. (2015). Cognitive reserve and lifestyle: moving towards preclinical Alzheimer’s disease. *Front. Aging Neurosci.* 7:134. 10.3389/fnagi.2015.00134 26321944 PMC4530312

[B7] Berner J. Comijs H. Elmståhl S. Welmer A.-K. Sanmartin Berglund J. Anderberg P. (2019). Maintaining cognitive function with internet use: a two-country, six-year longitudinal study. *Int. Psychoger.* 31 929–936. 10.1017/S1041610219000668

[B8] Brayne C. (2007). The elephant in the roomhealthy brains in later life, epidemiology and public health. *Nat. Rev. Neurosci.* 8 233–239. 10.1038/nrn2091 17299455

[B9] Briones T. L. Klintsova A. Y. Greenough W. T. (2004). Stability of synaptic plasticity in the adult rat visual cortex induced by complex environment exposure. *Brain Res.* 1018 130–135. 10.1016/j.brainres.2004.06.001 15262214

[B10] Brown C. L. Gibbons L. E. Kennison R. F. Robitaille A. Lindwall M. Mitchell M. B. (2012). Social activity and cognitive functioning over time: a coordinated analysis of four longitudinal studies. *J. Aging Res.* 2012 1–12. 10.1155/2012/287438 22991665 PMC3444000

[B11] Cadar D. Lassale C. Davies H. Llewellyn D. J. Batty G. D. Steptoe A. (2018). Individual and area-based socioeconomic factors associated with dementia incidence in England: evidence from a 12-year follow-up in the English longitudinal study of ageing. *JAMA Psychiatry* 75 723–732. 10.1001/jamapsychiatry.2018.1012 29799983 PMC6145673

[B12] Cassady K. Gagnon H. Lalwani P. Simmonite M. Foerster B. Park D. (2019). Sensorimotor network segregation declines with age and is linked to GABA and to sensorimotor performance. *Neuroimage* 186 234–244. 10.1016/j.neuroimage.2018.11.008 30414983 PMC6338503

[B13] Chan D. Shafto M. Kievit R. Matthews F. Spink M. Valenzuela M. (2018). Lifestyle activities in mid-life contribute to cognitive reserve in late-life, independent of education, occupation, and late-life activities. *Neurobiol. Aging* 70 180–183. 10.1016/J.NEUROBIOLAGING.2018.06.012 30025291 PMC6805221

[B14] Cheng S.-T. T. (2016). Cognitive reserve and the prevention of dementia: the role of physical and cognitive activities. *Curr. Psychiatry Rep.* 18:85. 10.1007/s11920-016-0721-2 27481112 PMC4969323

[B15] Cornelis M. C. Wang Y. Holland T. Agarwal P. Weintraub S. Morris M. C. (2019). Age and cognitive decline in the UK biobank. *PLoS One* 14:e0213948. 10.1371/journal.pone.0213948 30883587 PMC6422276

[B16] Cox S. R. Lyall D. M. Ritchie S. J. Bastin M. E. Harris M. A. Buchanan C. R. (2019). Associations between vascular risk factors and brain MRI indices in UK biobank. *Eur. Heart J.* 40 2290–2300. 10.1093/eurheartj/ehz100 30854560 PMC6642726

[B17] Demirakca T. Cardinale V. Dehn S. Ruf M. Ende G. (2016). The exercising brain: changes in functional connectivity induced by an integrated multimodal cognitive and whole-body coordination training. *Neural. Plast.* 2016:8240894. 10.1155/2016/8240894 26819776 PMC4706972

[B18] Dobromyslin V. I. Salat D. H. Fortier C. B. Leritz E. C. Beckmann C. F. Milberg W. P. (2012). Distinct functional networks within the cerebellum and their relation to cortical systems assessed with independent component analysis. *Neuroimage* 60 2073–2085. 10.1016/j.neuroimage.2012.01.139 22342804 PMC3549335

[B19] Domingos C. Pêgo J. M. Santos N. C. (2021). Effects of physical activity on brain function and structure in older adults: a systematic review. *Behav. Brain Res.* 402:113061. 10.1016/j.bbr.2020.113061 33359570

[B20] Ell S. Helie S. Hutchinson S. (2011). “Contributions of the putamen to cognitive function,” in *Horizons in Neuroscience Research*, (Nova Science Publishers Inc), 29–52. Available online at: http://ahuman.org/svn/ahengine/research/articles/Biological/2011-Contributions-of-putamen-to-cognitive-function.pdf (accessed July 29, 2019).

[B21] Erickson K. I. Hillman C. Stillman C. M. Ballard R. M. Bloodgood B. Conroy D. E. (2019). Physical activity, cognition, and brain outcomes: a review of the 2018 physical activity guidelines. *Med. Sci. Sports Exerc.* 51 1242–1251. 10.1249/MSS.0000000000001936 31095081 PMC6527141

[B22] Erickson K. I. Leckie R. L. Weinstein A. M. (2014). Physical activity, fitness, and gray matter volume. *Neurobiol. Aging* 35 S20–S28. 10.1016/j.neurobiolaging.2014.03.034 24952993 PMC4094356

[B23] Evans I. E. M. Martyr A. Collins R. Brayne C. Clare L. (2018). Social isolation and cognitive function in later life: a systematic review and meta-analysis. *J. Alzheimer’s Dis.* 70 S119–S144. 10.3233/JAD-180501 30372678 PMC6700717

[B24] Fancourt D. Steptoe A. Cadar D. (2018). Cultural engagement and cognitive reserve: museum attendance and dementia incidence over a 10-year period. *Br. J. Psychiatry* 213 661–663. 10.1192/bjp.2018.129 30025547 PMC6429239

[B25] Fjell A. M. Walhovd K. B. (2010). Structural brain changes in aging: courses, causes and cognitive consequences. *Rev. Neurosci.* 21 187–221. 10.1515/REVNEURO.2010.21.3.187 20879692

[B26] Foubert-Samier A. Catheline G. Amieva H. Dilharreguy B. Helmer C. Allard M. (2012). Education, occupation, leisure activities, and brain reserve: a population-based study. *Neurobiol. Aging* 33 423.e15-25. 10.1016/j.neurobiolaging.2010.09.023 21074901

[B27] Fratiglioni L. Paillard-Borg S. Winblad B. (2004). An active and socially integrated lifestyle in late life might protect against dementia. *Lancet Neurol.* 3 343–353. 10.1016/S1474-4422(04)00767-7 15157849

[B28] Fry A. Littlejohns T. J. Sudlow C. Doherty N. Adamska L. Sprosen T. (2017). Comparison of *Sociodemographic* and health-related characteristics of uk biobank participants with those of the general population. *Am. J. Epidemiol.* 186 1026–1034. 10.1093/aje/kwx246 28641372 PMC5860371

[B29] Galvin J. E. Tolea M. I. Chrisphonte S. (2021). The cognitive & leisure activity scale (CLAS): a new measure to quantify cognitive activities in older adults with and without cognitive impairment. Alzheimer’s Dement. *Transl. Res. Clin. Interv.* 7:e12134. 10.1002/trc2.12134 33816759 PMC8012243

[B30] Gow A. J. Pattie A. Deary I. J. (2017). Lifecourse activity participation from early, mid, and later adulthood as determinants of cognitive aging: the lothian birth cohort 1921. *J. Gerontol. B. Psychol. Sci. Soc. Sci.* 72 25–37. 10.1093/geronb/gbw124 27974473 PMC5156497

[B31] Grisolía J. M. Willis K. Wymer C. Law A. (2010). Social engagement and regional theatre: patterns of theatre attendance. *Cult. Trends* 19 225–244. 10.1080/09548963.2010.495277

[B32] Habas C. Kamdar N. Nguyen D. Prater K. Beckmann C. F. Menon V. (2009). Distinct cerebellar contributions to intrinsic connectivity networks. *J. Neurosci.* 29 8586–8594. 10.1523/JNEUROSCI.1868-09.2009 19571149 PMC2742620

[B33] James B. D. Glass T. A. Caffo B. Bobb J. F. Davatzikos C. Yousem D. (2012). Association of social engagement with brain volumes assessed by structural MRI. *J. Aging Res.* 2012:512714. 10.1155/2012/512714 22997582 PMC3446736

[B34] Jung C. K. E. Herms J. (2014). Structural dynamics of dendritic spines are influenced by an environmental enrichment: an in vivo imaging study. *Cereb. Cortex* 24 377–384. 10.1093/cercor/bhs317 23081882

[B35] Kelly M. E. Loughrey D. Lawlor B. A. Robertson I. H. Walsh C. Brennan S. (2014). The impact of cognitive training and mental stimulation on cognitive and everyday functioning of healthy older adults: a systematic review and meta-analysis. *Ageing Res. Rev.* 15 28–43. 10.1016/J.ARR.2014.02.004 24607830

[B36] Krell-Roesch J. Syrjanen J. A. Vassilaki M. Machulda M. M. Mielke M. M. Knopman D. S. (2019). Quantity and quality of mental activities and the risk of incident mild cognitive impairment. *Neurology* 93 e548–e558. 10.1212/WNL.0000000000007897 31292224 PMC6710000

[B37] Krell-Roesch J. Vemuri P. Pink A. Roberts R. O. Stokin G. B. Mielke M. M. (2017). Association between mentally stimulating activities in late life and the outcome of incident mild cognitive impairment, with an analysis of the APOE ε4 genotype. *JAMA Neurol.* 74:332. 10.1001/jamaneurol.2016.3822 28135351 PMC5473779

[B38] Kueider A. M. Parisi J. M. Gross A. L. Rebok G. W. (2012). Computerized cognitive training with older adults: a systematic review. *PLoS One* 7:e40588. 10.1371/journal.pone.0040588 22792378 PMC3394709

[B39] Kuiper J. S. Zuidersma M. Zuidema S. U. Burgerhof J. G. M. Stolk R. P. Oude Voshaar R. C. (2016). Social relationships and cognitive decline: a systematic review and meta-analysis of longitudinal cohort studies. *Int. J. Epidemiol.* 30:dyw089. 10.1093/ije/dyw089 27272181

[B40] Lemaitre H. Goldman A. L. Sambataro F. Verchinski B. A. Meyer-Lindenberg A. Weinberger D. R. (2012). Normal age-related brain morphometric changes: nonuniformity across cortical thickness, surface area and gray matter volume? *Neurobiol. Aging* 33 617.e1–617.e9. 10.1016/j.neurobiolaging.2010.07.013 20739099 PMC3026893

[B41] Livingston G. Huntley J. Sommerlad A. Ames D. Ballard C. Banerjee S. (2020). Dementia prevention, intervention, and care: 2020 report of the lancet commission. *Lancet* 396 413–446. 10.1016/S0140-6736(20)30367-6 32738937 PMC7392084

[B42] Mak H. Coulter R. Fancourt D. (2020). Patterns of social inequality in arts and cultural participation: findings from a nationally representative sample of adults living in the united kingdom of great Britain and Northern Ireland. *Public Heal. Panor.* 6 55–68. PMC7613128 35874800

[B43] Mallek M. Benguigui N. Dicks M. Thouvarecq R. (2017). Sport expertise in perception–action coupling revealed in a visuomotor tracking task. *Eur. J. Sport Sci.* 17 1270–1278. 10.1080/17461391.2017.1375014 28961061

[B44] Matyas N. Keser Aschenberger F. Wagner G. Teufer B. Auer S. Gisinger C. (2019). Continuing education for the prevention of mild cognitive impairment and Alzheimer’s-type dementia: a systematic review and overview of systematic reviews. *BMJ Open* 9:e027719. 10.1136/bmjopen-2018-027719 31270114 PMC6609120

[B45] Miller K. L. Alfaro-Almagro F. Bangerter N. K. Thomas D. L. Yacoub E. Xu J. (2016). Multimodal population brain imaging in the UK biobank prospective epidemiological study. *Nat. Neurosci.* 19 1523–1536. 10.1038/nn.4393 27643430 PMC5086094

[B46] Mitchell M. B. Cimino C. R. Benitez A. Brown C. L. Gibbons L. E. Kennison R. F. (2012). Cognitively stimulating activities: effects on cognition across four studies with up to 21 years of longitudinal data. *J. Aging Res.* 2012 1–12. 10.1155/2012/461592 23024862 PMC3449118

[B47] Mortimer J. A. Ding D. Borenstein A. R. DeCarli C. Guo Q. Wu Y. (2012). Changes in brain volume and cognition in a randomized trial of exercise and social interaction in a community-based sample of non-demented Chinese elders. *J. Alzheimers. Dis.* 30 757–766. 10.3233/JAD-2012-120079 22451320 PMC3788823

[B48] Nguyen L. Murphy K. Andrews G. (2019). Immediate and long-term efficacy of executive functions cognitive training in older adults: a systematic review and meta-analysis. *Psychol. Bull.* 145 698–733. 10.1037/bul0000196 30998045

[B49] Nucci M. Mapelli D. Mondini S. (2012). Cognitive reserve index questionnaire (CRIQ): a new instrument for measuring cognitive reserve. *Aging Clin. Exp. Res.* 24 218–226. 10.3275/7800 21691143

[B50] Nyberg L. Lö Vdé N M. Riklund K. Lindenberger U. Bä Ckman L. Lövdén M. (2012). Memory aging and brain maintenance. *Trends Cogn. Sci.* 16 292–305. 10.1016/j.tics.2012.04.005 22542563

[B51] Okuda H. Tatsumi K. Makinodan M. Yamauchi T. Kishimoto T. Wanaka A. (2009). Environmental enrichment stimulates progenitor cell proliferation in the amygdala. *J. Neurosci. Res.* 87 3546–3553. 10.1002/jnr.22160 19565652

[B52] Paillard-Borg S. Wang H.-X. Winblad B. Fratiglioni L. (2009). Pattern of participation in leisure activities among older people in relation to their health conditions and contextual factors: a survey in a Swedish urban area. *Ageing Soc.* 29 803–821. 10.1017/S0144686X08008337

[B53] Patterson C. (2018). *World Alzheimer Report 2018.*

[B54] Prince M. M. Wimo A. Guerchet M. Gemma-Claire A. Wu Y.-T. Prina M. (2015). *World Alzheimer Report 2015 The Global Impact of Dementia An Analysis of Prevalence, Incidence, Cost and Trends.* London. Available online at: https://www.alz.co.uk/research/WorldAlzheimerReport2015.pdf (accessed April 2, 2017).

[B55] Raz N. Lindenberger U. Rodrigue K. M. Kennedy K. M. Head D. Williamson A. (2005). Regional brain changes in aging healthy adults: general trends, individual differences and modifiers. *Cereb. Cortex* 15 1676–1689. 10.1093/cercor/bhi044 15703252

[B56] Seider T. R. Fieo R. A. O’Shea A. Porges E. C. Woods A. J. Cohen R. A. (2016). Cognitively engaging activity is associated with greater cortical and subcortical volumes. *Front. Aging Neurosci.* 8:1–10. 10.3389/fnagi.2016.00094 27199740 PMC4852201

[B57] Sexton C. E. Betts J. F. Demnitz N. Dawes H. Ebmeier K. P. Johansen-Berg H. (2016). A systematic review of MRI studies examining the relationship between physical fitness and activity and the white matter of the ageing brain. *Neuroimage* 131 81–90. 10.1016/j.neuroimage.2015.09.071 26477656 PMC4851455

[B58] Sexton C. E. Mackay C. E. Ebmeier K. P. (2013). A systematic review and meta-analysis of magnetic resonance imaging studies in late-life depression. *Am. J. Geriatr. Psychiatry* 21 184–195. 10.1016/j.jagp.2012.10.019 23343492

[B59] Smith J. C. Nielson K. A. Woodard J. L. Seidenberg M. Durgerian S. Antuono P. (2011). Interactive effects of physical activity and APOE-ε4 on BOLD semantic memory activation in healthy elders. *Neuroimage* 54 635–644. 10.1016/j.neuroimage.2010.07.070 20691792 PMC2962671

[B60] Smith S. M. Fox P. T. Miller K. L. Glahn D. C. Fox P. M. Mackay C. E. (2009). Correspondence of the brain’s functional architecture during activation and rest. *Proc. Natl. Acad. Sci. U.S.A.* 106 13040–13045. 10.1073/pnas.0905267106 19620724 PMC2722273

[B61] Stephen R. Liu Y. Ngandu T. Antikainen R. Hulkkonen J. Koikkalainen J. (2019). Brain volumes and cortical thickness on MRI in the finnish geriatric intervention study to prevent cognitive impairment and disability (FINGER). *Alzheimers. Res. Ther.* 11:53. 10.1186/s13195-019-0506-z 31164160 PMC6549301

[B62] Stern Y. (2012). Cognitive reserve in ageing and Alzheimer’s disease. *Lancet Neurol.* 11 1006–1012. 10.1016/S1474-4422(12)70191-6 23079557 PMC3507991

[B63] Stillman C. M. Donofry S. D. Erickson K. I. (2019). *Exercise, Fitness and the Aging Brain: A Review of Functional Connectivity in Aging.* Available online at: http://www.archivesofpsychology.org (accessed June 3, 2021).

[B64] Tun P. A. Lachman M. E. (2010). The association between computer use and cognition across adulthood: use it so you won’t lose it? *Psychol. Aging* 25:560. 10.1037/A0019543 20677884 PMC3281759

[B65] Valenzuela M. J. Sachdev P. Wen W. Chen X. Brodaty H. (2008). Lifespan mental activity predicts diminished rate of hippocampal atrophy. *PLoS One* 3:1–6. 10.1371/journal.pone.0002598 18612379 PMC2440814

[B66] Wang H.-X. Xu W. Pei J.-J. J. (2012). Leisure activities, cognition and dementia. *Biochim. Biophys. Acta Mol. Basis Dis.* 1822 482–491. 10.1016/j.bbadis.2011.09.002 21930203

[B67] Wassenaar T. M. Yaffe K. van der Werf Y. D. Sexton C. E. (2019). Associations between modifiable risk factors and white matter of the aging brain: insights from diffusion tensor imaging studies. *Neurobiol. Aging* 80 56–70. 10.1016/J.NEUROBIOLAGING.2019.04.006 31103633 PMC6683729

[B68] Wilson R. S. Barnes L. L. Krueger K. R. Hoganson G. Bienias J. L. (2005). Early and late life cognitive activity and cognitive systems in old age. *J. Int. Neuropsychol. Soc.* 11 400–407. 10.1017/S1355617705050459 16209420

[B69] Wilson R. S. Bennett D. A. Beckett L. A. Morris M. C. Gilley D. W. Bienias J. L. (1999). Cognitive activity in older persons from a geographically defined population. *J. Gerontol. B. Psychol. Sci. Soc. Sci.* 54 155–160. 10.1093/geronb/54B.3.P155 10363036

[B70] Wilson R. Barnes L. Bennett D. (2003). Assessment of lifetime participation in cognitively stimulating activities. *J. Clin. Exp. Neuropsychol.* 25 634–642. 10.1076/jcen.25.5.634.14572 12815501

[B71] World Health Organization (2015). *World Report on Ageing and Health.* Luxembourg: World Health Organization.

[B72] Yates L. A. Ziser S. Spector A. Orrell M. (2016). Cognitive leisure activities and future risk of cognitive impairment and dementia: systematic review and meta-analysis. *Int. Psychogeriatr.* 28 1–16. 10.1017/S1041610216001137 27502691

[B73] Yip A. G. Brayne C. Matthews F. E., and Mrc Cognitive Function and Ageing Study. (2006). Risk factors for incident dementia in England and Wales: the medical research council cognitive function and ageing study. a population-based nested case–control study. *Age Ageing* 35 154–160. 10.1093/ageing/afj030 16414964

[B74] Zhao Y. Y. Shi X. Y. Qiu X. Zhang L. Lu W. Yang S. (2011). Enriched environment increases the total number of CNPase positive cells in the corpus callosum of middle-aged rats. *Acta Neurobiol. Exp.* 71 322–330. 10.55782/ane-2011-1854 22068741

