# Supplementary material for: Leisure Activities and Their Relationship With MRI Measures of Brain Structure, Functional Connectivity, and Cognition in the UK Biobank Cohort
Source: Front Aging Neurosci. 2021 Nov 16;13:734866. doi: 10.3389/fnagi.2021.734866 (PMC8635062; doi:10.3389/fnagi.2021.734866)
Supplement: Supplementary file 1 [file Data_Sheet_1.docx]

**SUPPLEMENTARY MATERIALS**

**Figure S1.** A timeline of the study assessments and variables of interest examined in the current analyses.

**Leisure Activities**

**Leisure Activities**

**MRI and**

**Cognitive outcomes**

2006 - 2010

2014 +

**Baseline**

**Follow-up**

**Abbreviations-** MRI = Magnetic resonance imaging.

**Figure S2.** Flowchart of participant selection and exclusion.

**SUPPLEMENTARY METHODS**

**Activity measures**

Respondents were required to indicate from a list of activities, those that were undertaken on a weekly (or more frequent) basis. The activities measured consisted of going to a pub or social club, participating in a religious activity, attending adult education classes and going to a sports club or visiting the gym. A fifth item, which asked individuals to indicate whether they regularly participated in “other group activities” was excluded due to the ambiguity of the question posed. Responses on these four items were coded as a “1” for weekly participation, or a “0” for irregular/ no participation.

Separately, respondents were also asked to indicate how frequently they received or made friend or family visits, with possible answers consisting of: “almost daily”, “2-4 times a week”, “about once a week”, “about once a month”, “once every few months” or “never or almost never”. Leisure-time computer use was included as another item, with participants asked to indicate the number of hours (per week) they committed to this activity in a typical day. To avoid biases introduced by differences in the granularity of our various activity scales, we binarized responses on these items. Specifically, we assigned a code of “0” for individuals who received or made friend and family visits at least once a week, and an assignment of “0” for those who visited friends and family less frequently than once a week. Weekly or more frequent family/friends were coded as “1”. Similarly, participants reporting 0 hours of daily computer use were coded as “0”, whereas those reporting any amount of daily use were grouped as weekly engagers (i.e., “1”). For all activities, answers corresponding to “do not know” or “prefer not to answer” were coded as missing data.

**Cognitive measures**

A cognitive battery designed specifically for UK Biobank was administered via a tablet. While the self-administered and unsupervised nature of these tests may call into question the reliability and validity of these tests, a recent study of 160 adults (Fawns-Ritchie and Deary, 2019) shows that the UK Biobank cognitive assessments have moderate-to-high test-retest reliability and moderate-to-high concurrent validity, as indicated by correlations between each of these measures with that of validated and standardized tests, delivered in a face-to-face setting. The selection of cognitive tests examined in the present study was guided by prior research (Chan et al., 2018; Cornelis et al., 2019). Some tests, for instance of language fluency, were only administered at the pilot phase of the UK Biobank, and so were excluded here. Overall, several previous studies have demonstrated that the cognitive tests examined here are sensitive to age-related differences in performance (Cornelis et al., 2019; Fawns-Ritchie and Deary, 2019).

The cognitive battery included a 13-item questionnaire Fluid Intelligence test. This questionnaire included reasoning/logic items that each had 5 multiple choice options. Participants were required to select one of these responses within a 2-minute period. For example, one of the items asked: “Relaxed means the opposite of?”, with the response options consisting of “calm”, “anxious”, “cool”, “worried” and “tense”. The outcome of this test was the total number of questions answered correctly.

Numeric and alphanumeric trail making tasks were also completed by a portion of the total sample. This task presented participants with 25 circles distributed across the screen, which contained either numbers ranging from 1-25 (numeric trail) or a mix of numbers and letters (alphanumeric trail). Participants were asked to connect the circles from the smallest to largest numbers in one task (numeric trail) or to alternate between numbers and letters in an ascending sequence in the other (alphanumeric trail). The outcomes on these tasks were the time taken to complete the trail, with lower values reflecting a faster completion time. Individuals who spent ≥ 250 seconds on these respective tests, were excluded (Hagenaars et al., 2018).

A digit span test was also administered. Individuals were presented with a sequence of digits, which they were instructed to recall in a reverse order (starting with the last digit first) after a short delay. Each time the sequence of digits was recalled correctly, the number of digits to be remembered increased by 1. The number began at 2 digits and increased to a maximum number of 12 digits. The test terminated as soon as an error on a given trial was made. The outcome here was the maximum number of digits recalled.

Another test, known as pairs matching, relied on the use of computerized cards. Here, participants were presented with a set of cards (organized into 3 rows, 4 columns) and were briefly shown the symbols that the cards contained before they were turned face down. Each card had a matching pair, with the task requiring respondents to match the six pairs of cards from memory while making as few errors as possible. The main outcome was the number of incorrect matches made.

The prospective memory test began with a screen containing four coloured symbols: a blue square, a pink star, a grey cross, and an orange circle. Text also accompanied the four symbols that informed participants that they would see the screen again at the end of the cognitive battery, and they would be asked to press the blue square. The text instructed participants that rather than touching the blue square (as the later instructions dictated), they should instead press the orange circle. The outcome was whether or not participants had responded corrected (i.e., touched the orange circle) on the first attempt.

A symbol digit matching task was also administered. In this task, individuals were presented with a set of grids with each box containing a symbol. Participants were required to match each symbol with a unique digit, with the correct symbol-digit matches detailed in a key that was available throughout the 1-minute response period. The outcome was the number of correct symbol-digit matches made (excluding the first 8 items that served as training material).

Finally, a simple reaction time task was also completed, which is analogous to the card game ‘Snap’. For this assessment, participants were presented with a pair of cards (total number of pairs = 12) and were required to press a button as quickly as possible whenever the symbols on the cards were matching. Four training trials were first completed, after which 7 recorded trials were administered (4 of which contained matching pairs). The key outcome was the mean response time (in seconds) across the 4 trials containing matching pairs.

**Demographics and health-related variables**

Educational level was assessed by asking respondents whether they possessed one or more of the following qualifications: “college or university degree” (= “5”), “NVQ or HND or HNC or equivalent” (= “4”), “other professional qualifications (e.g., nursing, teaching)” (= “4”), “A levels/AS levels or equivalent” (= “3”), “O levels/ GCSEs or equivalent” (= “2”) and “CSE or equivalent” (= “1”). From this information, we created a variable indicating the highest qualification earned. Occupational status was coded according to the Standard Occupational Classification, where participants were divided into one of nine occupational groups: “Manager and Senior Officials” (= “9”), “Professional Occupations” (= “8”), “Associate Professional and Technical Occupations” (= “7”), “Administrative and Secretarial Occupations” (= “6”), “Skilled Trades Occupations” (= “5”), “Personal Service Occupations” (= “4”), “Sales and Customer Service Occupations” (= “3”), “Process, plant and machine operatives” (= “2”) and “Elementary Occupations” (= “1”). An additional category, reflecting the lowest level of this variable included those who were retired, unemployed, looking after home and/ or family, unable to work because of sickness or disability, full or part time student (= “0”).

Frequency of alcohol intake (over the last year) was measured as “daily or almost daily” (= “5”), “three or four times a week” (= “4”), “once or twice a week” (= “3”), “one to three times a month” (= “2”), “special occasions only” (= “1”) or “never” (= “0”). Sleep duration was measured as the hours of sleep within an average 24-hour period. Body mass index (BMI) was calculated as: (weight [kilograms]/height [metres])^2^. Mean Arterial Pressure (MAP) was also calculated based on the systolic and diastolic indices measured with an electronic tool (systolic blood pressure + 2* diastolic blood pressure/3). Note that in the case where two measurements of diastolic/systolic measures were available, an average over these measures were created before calculating the MAP. If only a single assessment was available, then this was used for to calculate the MAP. Diagnoses of depressive (e.g., major depression) and anxiety disorders (e.g., social anxiety), coded according to the World Health Organization’s International Classification of Diseases manual (ICD-10), over the study period were also taken into account. Until April 2010, ICD-10 was used, with ICD-10 4^th^ edition used from April 2010 to date (Wu et al., 2019). An index of social isolation was further included (Shankar et al., 2013), assessed as the total number of individuals living in the household (alongside the participant).

**MRI Data Acquisition**

T1 images were acquired with a resolution of 1 x 1 x 1 mm and a field of view of 256 mm, in the sagittal plane with a 3D magnetization-prepared rapid gradient echo (MPRAGE). The inversion and repetition times were 880 ms and 2000 ms, respectively. Diffusion-weighted images were collected using a spin-echo echo planar imaging sequence, with 2 mm isotropic voxels, a 104 x 104 mm field of view, an echo time of 92 ms, repetition time of 3600 ms and a multiband acceleration factor of 3 (i.e., three slices acquired at a time (Miller et al., 2016). Five b0 images were collected, in addition to images acquired with two separate b-values (50x b = 1000 s/mm^2^ and 50x b = 2,0000 s/mm^2^), which amounted to 100 diffusion-encoding directions. T2-weighted FLAIR imaging was additionally acquired with 3D SPACE in the sagittal plane (resolution = 1.05 x 1 x 1 mm, field of view = 192 x 256 x 256 mm; inversion time = 1800 ms, repetition time = 5000 ms). Finally, resting-state fMRI images were acquired with a gradient echo-echo planar imaging (GE-EPI) using a multi-slice acceleration of 8 and a flip angle of 52° (2.4 x 2.4 x 2.4 mm voxels; field of view = 88 x 88 x 64 mm, 490 timepoints, repetition time = 0.745 seconds, echo time = 39 ms).

**MRI Data Pre-processing**

Measures of brain structure and functional connectivity, or Image Derived Phenotypes (IDPs), were generated using FMRIB’s Biobank Pipeline (version 1.0, (Alfaro-Almagro et al., 2018). For a detailed description of the imaging protocol and pre-processing steps, please see Alfaro-Almagro et al. ( 2018) and Smith et al. (2019).

The T1-weighted images were first defaced to anonymize the images. The size of field of view was then reduced to remove voxels containing non-brain tissue, using a combination of FSL’s Brain Extraction Tool (Smith, 2002); linear registration (Jenkinson et al., 2002; Jenkinson and Smith, 2001), and the MNI152 “nonlinear 6^th^ generation” standard space T1 template (<http://www.bic.mni.mcgill.ca/ServicesAtlases/ICBM152NLin6>). Gradient distortion correction was also applied at this point. Next, nonlinear registration was performed with FNIRT (Andersson et al., 2007) to calculate the T1-to-MNI152 warp transform, with a custom brain mask as a reference image. Using the inverse of the warp transform, the standard space brain mask was then transformed into each individual’s native T1 space in order to brain extract for each individual. FAST (Zhang et al., 2001) was subsequently applied to fulfil two objectives on the brain extracted T1 images: (1) segment the images based on tissue types (i.e., GM, WM and CSF) and (2) generate a bias-field corrected image. Partial volume estimates of GM from FAST were parcellated into 139 GM ROIs, using a combination of the Harvard-Oxford cortical and subcortical atlases (<https://fsl.fmrib.ox.ac.uk/fsl/fslwiki/Atlases>) and Diedrichsen cerebellar atlas (<http://www.diedrichsenlab.org/imaging/propatlas.htm>) to facilitate parcellation. FSL’s FIRST was also employed to extract volumetric estimates of key subcortical structures, including the hippocampus, amygdala, thalamus, pallidum, caudate, putamen (Patenaude et al., 2011). Note that the corresponding FAST-extracted ROIs for these subcortical regions were excluded. For a list of all of the regional GM IDPs examined in this study, see Appendix 5.2. Finally, in order to derive an estimate of head size (used as a co-variate in the present analyses), the pre-processed T1 images were separately submitted to a SIENAX (Structural Image Evaluation, using Normalisation, of Atrophy: Cross-sectional; (Smith et al., 2002).

For the raw diffusion-weighted images, EPI distortions and eddy currents/outlier slices were addressed with FSl’s topup (Andersson et al., 2003) and eddy, respectively (Andersson and Sotiropoulos, 2016, 2015). The next step was to apply gradient distortion correction (developed by HCP and FSL) to remove artefacts introduced by head motion. DTI fit (Basser et al., 1994) was applied to derive FA and MD images for each participant. The pre-processed diffusion-weighted images were subsequently submitted to a tractography-based analysis. Specifically, BEDPOSTX (Bayesian Estimation of Diffusion Parameters Obtained using Sampling Techniques, <http://fsl.fmrib.ox.ac.uk/fsl/fslwiki/FDT/UserGuide>) was used, which is a method for intra-voxel modelling of multi-fibre tract orientations with the capacity to estimate up to three fibre orientations within a voxel. The output of BEDPOSTX were then fed to PROBTRACKX (Behrens et al., 2007, 2003; Hernández et al., 2013; Jbabdi et al., 2012), a tool that conducts probabilistic tractography (implemented with a crossing-fibre model), which is currently able to map 27 major tracts using start/stop ROI masks defined by AutoPX (de Groot et al., 2013). The tracts included the cingulum bundle, thalamic radiations, longitudinal fasciculi with a full list available in the Appendix 5.3. As the results of both of these tools are in native space, non-linear transformations were applied to bring the results into 1mm standard MNI space. The IDPs made available after these pre-processing steps were the weighted-mean FA and MD for each of the 27 tracts. After defacing, the T2 FLAIR structural images were registered to the T1-weighted images using FLIRT (Greve and Fischl, 2009). The transforms generated were then used to register the FLAIR images to MNI space. An estimate of total WM hyperintensity volume was generated by feeding the T2-weighted FLAIR and T1-weighted images to the BIANCA tool developed by Griffanti et al. (Griffanti et al., 2016).

The pre-processing steps involved motion correction (via MCFLIRT, (Jenkinson et al., 2002), grand-mean intensity normalisation of the 3D dataset, high pass temporal filtering, EPI and GDC unwarping. ICA+FIX processing were applied to remove the presence of individual-level structured artefacts (Beckmann and Smith, 2004; Griffanti et al., 2014; Salimi-Khorshidi et al., 2014). The images were then brought into T1 space and then standard MNI space, using FLIRT (with a BBR cost function; Greve and Fischl, 2009). Next, low-dimensionality Group ICA (n. of components = 25) was applied to the pre-processed functional images of 4,162 individuals (Alfaro-Almagro et al., 2018) in order to derive group-level spatial maps of large-scale resting state networks. Twenty-one of these components were deemed to be ‘signal’ of interest (see Figure S3 for an overview of components; hand labelled by Melis Anatürk (MA), Sana Suri (SS) and Claire Sexton (CES) with disagreements resolved through discussion). These components were then submitted to a FSLNETS analysis. This tool was used to generate a 21x21 matrix (i.e., connectomes) representing the correlations (i.e., edge or connectivity) between pairs of large-scale functional networks (i.e., nodes). Partial correlations were used as they provide a more direct estimate of the connectivity between two nodes, with estimates adjusted for the connectivity of all other nodes in the connectome. Estimates of partial correlations were derived with an L2 Regularization (rho = 0.05 in the Ridge Regression option in FSL Nets). All values were transformed from Pearson’s correlations to z-statistics. Prior to the analysis, we used a method validated by prior studies (Shen et al., 2018; Smith et al., 2015) to improve the interpretability of our results. Specifically, values for individuals were multiplied by the sign of their mean edge value, in order to give an index of *absolute connectivity,* with higher values reflecting stronger connections. Head motion (derived from McFLIRT) represented the mean relative displacement acquired during the acquisition of functional images and was used here as a co-variate.

**FDR Corrections**FDR corrections were applied across imaging modalities. FDR corrections were applied separately for the analysis of cognitive tests, as this was conducted in a smaller sample to the main analytical group.

**Figure S3. Classifications of signal components from a 25-dimension ICA.**

**Abbreviations** – PCC = Posterior Cingulate Cortex.

**Figure S3 continued. Classifications of signal components from a 25-dimension ICA.**

| **Table S1. Comparisons between included and excluded individuals.** Bold text indicates comparisons that survived multiple comparison  corrections. | | | | | |
| --- | --- | --- | --- | --- | --- |
| **Dependent variable** | **Included** | **Excluded** | **Test-statistic** | **p-value** | **FDR q-value** |
| *MRI sample* |  |  |  |  |  |
| **Age at baseline (years), mean ± SD** | **56.39 ± 7.31** | **54.57 ± 7.5** | **t = -18.29** | **1.06e-73** | **1.76e-73** |
| **% female** | **4897 (54.5%)** | **4897 (51.1%)** | **χ2 = 25.93** | **3.55e-07** | **4.44e-07** |
| **Educational level, median (IQR)** | **5 (1)** | **4 (2)** | **W = 71598114** | **1.35e-93** | **3.38e-93** |
| **Occupational status, median (IQR)** | **8 (2)** | **7 (8)** | **W = 54761450** | **7.18e-127** | **3.59e-126** |
| % ICD-10 diagnosis of Depression/ Anxiety | 51 (0.7%) | 181 (0.8%) | χ2 = 0.22 | 0.64 | 0.64 |
| *Cognitive sample* |  |  |  |  |  |
| Age at baseline (years) | 55.08 ± 7.3 | 54.99 ± 7.5 | t = -0.48 | 0.63 | 0.64 |
| **% female** | **951 (54.8%)** | **14,984 (51.7%)** | **χ2 = 6.59** | **1.03e-02** | **1.71e-02** |
| **Educational level, median (IQR)** | **5 (1)** | **4 (2)** | **W =**  **20951382** | **3.48e-35** | **1.74e-34** |
| **Occupational status, median (IQR)** | **8 (2)** | **7 (8)** | **W =**  **17564356** | **4.00e-32** | **9.99e-32** |
| % ICD-10 diagnosis of Depression/ Anxiety | 15 (0.7%) | 217 (0.9%) | χ2 = 0.3 | 0.59 | 0.63 |
| **Abbreviations-** FDR = False Discovery Rate, ICD = International Classification of Diseases, SD = standard deviation.  **Note:** Welch’s t-tests were used for continuous variables (due to unequal variances between included and excluded participants), while chi-squared test was used for  binary variables. Wilcoxon’s rank sum test was used for ranked ordinal variables (i.e., educational level and occupational status). | | | | | |

**Supplementary References**

Alfaro-Almagro, F., Jenkinson, M., Bangerter, N.K., Andersson, J.L.R., Griffanti, L., Douaud, G., Sotiropoulos, S.N., Jbabdi, S., Hernandez-Fernandez, M., Vallee, E., Vidaurre, D., Webster, M., McCarthy, P., Rorden, C., Daducci, A., Alexander, D.C., Zhang, H., Dragonu, I., Matthews, P.M., Miller, K.L., Smith, S.M., 2018. Image processing and Quality Control for the first 10,000 brain imaging datasets from UK Biobank. Neuroimage 166, 400–424. doi:10.1016/J.NEUROIMAGE.2017.10.034

Andersson, J.L.R., Jenkinson, M., Smith, S., Jenkinson, M., SMITH, S., Andersson, J.L.R., Andersson, J., Smith, P.A.E.S., Andersson, J.L., Smith, S.W., 2007. Non-Linear Registration aka Spatial Normalisation FMRIB Technial Report TR07JA2.

Andersson, J.L.R., Skare, S., Ashburner, J., 2003. How to correct susceptibility distortions in spin-echo echo-planar images: application to diffusion tensor imaging. Neuroimage 20, 870–888. doi:10.1016/S1053-8119(03)00336-7

Andersson, J.L.R., Sotiropoulos, S.N., 2016. An integrated approach to correction for off-resonance effects and subject movement in diffusion MR imaging. Neuroimage 125, 1063–1078. doi:10.1016/j.neuroimage.2015.10.019

Andersson, J.L.R., Sotiropoulos, S.N., 2015. Non-parametric representation and prediction of single- and multi-shell diffusion-weighted MRI data using Gaussian processes. Neuroimage 122, 166–176. doi:10.1016/j.neuroimage.2015.07.067

Basser, P.J., Mattiello, J., LeBihan, D., 1994. MR diffusion tensor spectroscopy and imaging. Biophys. J. 66, 259–267. doi:10.1016/S0006-3495(94)80775-1

Beckmann, C.F., Smith, S.M., 2004. Probabilistic Independent Component Analysis for Functional Magnetic Resonance Imaging. IEEE Trans. Med. Imaging 23, 137–152. doi:10.1109/TMI.2003.822821

Behrens, T.E.J., Berg, H.J., Jbabdi, S., Rushworth, M.F.S., Woolrich, M.W., 2007. Probabilistic diffusion tractography with multiple fibre orientations: What can we gain? Neuroimage 34, 144–155. doi:10.1016/J.NEUROIMAGE.2006.09.018

Behrens, T.E.J., Woolrich, M.W., Jenkinson, M., Johansen-Berg, H., Nunes, R.G., Clare, S., Matthews, P.M., Brady, J.M., Smith, S.M., 2003. Characterization and propagation of uncertainty in diffusion-weighted MR imaging. Magn. Reson. Med. 50, 1077–1088. doi:10.1002/mrm.10609

Chan, D., Shafto, M., Kievit, R., Matthews, F., Spink, M., Valenzuela, M., Henson, R.N., 2018. Lifestyle activities in mid-life contribute to cognitive reserve in late-life, independent of education, occupation, and late-life activities. Neurobiol. Aging 70, 180–183. doi:10.1016/J.NEUROBIOLAGING.2018.06.012

Cornelis, M.C., Wang, Y., Holland, T., Agarwal, P., Weintraub, S., Morris, M.C., 2019. Age and cognitive decline in the UK Biobank. PLoS One 14, e0213948. doi:10.1371/journal.pone.0213948

de Groot, M., Vernooij, M.W., Klein, S., Ikram, M.A., Vos, F.M., Smith, S.M., Niessen, W.J., Andersson, J.L.R., 2013. Improving alignment in Tract-based spatial statistics: Evaluation and optimization of image registration. Neuroimage 76, 400–411. doi:10.1016/j.neuroimage.2013.03.015

Fawns-Ritchie, C., Deary, I., 2019. Reliability and validity of the UK Biobank cognitive tests. doi:10.1101/19002204

Greve, D.N., Fischl, B., 2009. Accurate and robust brain image alignment using boundary-based registration. Neuroimage 48, 63–72. doi:10.1016/j.neuroimage.2009.06.060

Griffanti, L., Salimi-Khorshidi, G., Beckmann, C.F., Auerbach, E.J., Douaud, G., Sexton, C.E., Zsoldos, E., Ebmeier, K.P., Filippini, N., Mackay, C.E., Moeller, S., Xu, J., Yacoub, E., Baselli, G., Ugurbil, K., Miller, K.L., Smith, S.M., 2014. ICA-based artefact removal and accelerated fMRI acquisition for improved resting state network imaging. Neuroimage 95, 232–247.

Griffanti, L., Zamboni, G., Khan, A., Li, L., Bonifacio, G., Sundaresan, V., Schulz, U.G., Kuker, W., Battaglini, M., Rothwell, P.M., Jenkinson, M., 2016. BIANCA (Brain Intensity AbNormality Classification Algorithm): A new tool for automated segmentation of white matter hyperintensities. Neuroimage 141, 191–205. doi:10.1016/j.neuroimage.2016.07.018

Hagenaars, S.P., Cox, S.R., Hill, W.D., Davies, G., Liewald, D.C.M., Harris, S.E., McIntosh, A.M., Gale, C.R., Deary, I.J., Deary, I.J., 2018. Genetic contributions to Trail Making Test performance in UK Biobank. Mol. Psychiatry 23, 1575–1583. doi:10.1038/mp.2017.189

Hernández, M., Guerrero, G.D., Cecilia, J.M., García, J.M., Inuggi, A., Jbabdi, S., Behrens, T.E.J., Sotiropoulos, S.N., 2013. Accelerating Fibre Orientation Estimation from Diffusion Weighted Magnetic Resonance Imaging Using GPUs. PLoS One 8, e61892. doi:10.1371/journal.pone.0061892

Jbabdi, S., Sotiropoulos, S.N., Savio, A.M., Graña, M., Behrens, T.E.J., 2012. Model-based analysis of multishell diffusion MR data for tractography: How to get over fitting problems. Magn. Reson. Med. 68, 1846–1855. doi:10.1002/mrm.24204

Jenkinson, M., Bannister, P., Brady, M., Smith, S., 2002. Improved optimization for the robust and accurate linear registration and motion correction of brain images. Neuroimage 17, 825–41.

Jenkinson, M., Smith, S., 2001. A global optimisation method for robust affine registration of brain images. Med. Image Anal. 5, 143–56.

Miller, K.L., Alfaro-Almagro, F., Bangerter, N.K., Thomas, D.L., Yacoub, E., Xu, J., Bartsch, A.J., Jbabdi, S., Sotiropoulos, S.N., Andersson, J.L.R., Griffanti, L., Douaud, G., Okell, T.W., Weale, P., Dragonu, I., Garratt, S., Hudson, S., Collins, R., Jenkinson, M., Matthews, P.M., Smith, S.M., 2016. Multimodal population brain imaging in the UK Biobank prospective epidemiological study. Nat. Neurosci. 19, 1523–1536. doi:10.1038/nn.4393

Patenaude, B., Smith, S.M., Kennedy, D.N., Jenkinson, M., 2011. A Bayesian model of shape and appearance for subcortical brain segmentation. Neuroimage 56, 907–22. doi:10.1016/j.neuroimage.2011.02.046

Salimi-Khorshidi, G., Douaud, G., Beckmann, C.F., Glasser, M.F., Griffanti, L., Smith, S.M., 2014. Automatic denoising of functional MRI data: Combining independent component analysis and hierarchical fusion of classifiers. Neuroimage 90, 449–468. doi:10.1016/j.neuroimage.2013.11.046

Shankar, A., Hamer, M., McMunn, A., Steptoe, A., 2013. Social Isolation and Loneliness. Psychosom. Med. 75, 161–170. doi:10.1097/PSY.0b013e31827f09cd

Shen, X., Cox, S.R., Adams, M.J., Howard, D.M., Lawrie, S.M., Ritchie, S.J., Bastin, M.E., Deary, I.J., McIntosh, A.M., Whalley, H.C., 2018. Resting-State Connectivity and Its Association With Cognitive Performance, Educational Attainment, and Household Income in the UK Biobank. Biol. Psychiatry Cogn. Neurosci. Neuroimaging 3, 878–886. doi:10.1016/J.BPSC.2018.06.007

Smith, R.X., Jann, K., Ances, B., Wang, D.J.J., 2015. Wavelet-based regularity analysis reveals recurrent spatiotemporal behavior in resting-state fMRI. Hum. Brain Mapp. 36, 3603–3620.

Smith, S.M., 2002. Fast robust automated brain extraction. Hum. Brain Mapp. 17, 143–155. doi:10.1002/hbm.10062

Smith, S.M., Alfaro-Almagro, F., Miller, K.L., 2019. UK Biobank Brain Imaging Documentation.

Smith, S.M., Zhang, Y., Jenkinson, M., Chen, J., Matthews, P.M., Federico, A., De Stefano, N., 2002. Accurate, robust, and automated longitudinal and cross-sectional brain change analysis. Neuroimage 17, 479–89.

Wu, P., Gifford, A., Meng, X., Li, X., Campbell, H., Varley, T., Zhao, J., Carroll, R., Bastarache, L., Denny, J.C., Theodoratou, E., Wei, W.-Q., 2019. Developing and Evaluating Mappings of ICD-10 and ICD-10-CM Codes to PheCodes. bioRxiv 462077. doi:10.1101/462077

Zhang, Y., Brady, M., Smith, S., 2001. Segmentation of brain MR images through a hidden Markov random field model and the expectation-maximization algorithm. IEEE Trans. Med. Imaging 20, 45–57. doi:10.1109/42.906424
